# Immunoassay of *S*-adenosylmethionine and *S*-adenosylhomocysteine: the methylation index as a biomarker for disease and health status

**DOI:** 10.1186/s13104-016-2296-8

**Published:** 2016-11-28

**Authors:** Xiujuan Hao, Yan Huang, Ming Qiu, Chunlin Yin, Huiming Ren, Hongjie Gan, Huijun Li, Yaxia Zhou, Jiazhi Xia, Wenting Li, Lijuan Guo, Isaac A. Angres

**Affiliations:** 1 Arthus Biosystems, 2600 Hilltop Dr., Richmond, CA 94806 USA; 2 Hunan SkyWorld Biotechnologies Co. Ltd, 9 Panpan Rd. Changsha Economic and Technological Development Zone, Changsha, Hunan 410100 China; 3 Department of Infectious Diseases, Xiangya Hospital, Central South University Hospital, Changsha, China; 4 Department of Cardiology, Xuanwu Hospital, Capital Medical University, Beijing, 100053 China; 5 Changsha Blood Center, Changsha, 410000 China; 6 Lanwei Clinical Laboratories Co. Ltd., 268 Xinxing Rd. International Enterprise Center Building 9, Suite 301, Yuhua District, Changsha, 410000 China

**Keywords:** Antibody, Immunoassay, Methylation index, *S*-Adenosylmethionine, *S*-Adenosylhomocysteine, Diseases, Biomarker, Competitive ELISA, Conjugates

## Abstract

**Background:**

*S*-Adenosylmethionine (SAM) and *S*-adenosylhomocysteine (SAH) are relevant to a variety of diseases. Previous reports that quantified SAM and SAH were based on HPLC or LC–MS/MS. No antibody against SAM has been generated, and the antibody against SAH cannot be used with blood samples. Immunoassays have not been used to measure SAM and SAH. In this study, ELISA was used to measure blood SAM and SAH levels.

**Results:**

Specific antibodies against SAM were produced for the first time using a stable analog as the antigen. The monoclonal antibodies against SAM and SAH were characterized. No cross-reactivity was detected for the analyzed analogs. For the anti-SAM antibodies, the ELISA sensitivity was ~2 nM, and the affinity was 7.29 × 10^10^ L/mol. For the anti-SAH antibodies, the sensitivity was ~15 nM, and the affinity was 2.79 × 10^8^ L/mol. Using high-quality antibodies against SAM and SAH, immunoassays for the detection of SAM and SAH levels in blood and tissue samples were developed. Clinical investigations using immunoassays to measure SAM, SAH and the methylation index (MI) in normal and diseased samples indicated that (1) the SAM level is age and gender dependent; (2) the SAM level is associated with the severity of liver diseases, inflammatory reactions and other diseases; and (3) the methylation index (MI) is significantly reduced in many diseases and may serve as a screening biomarker to identify potentially unfavorable health conditions.

**Conclusion:**

It is possible to generate antibodies against active small biomolecules with weak immunogenicity, such as SAM and SAH, using traditional hybridoma technology. The antigens and antibodies described here will contribute to the development of immunoassays to measure SAM, SAH and related molecules. These assays enable the MI to be measured specifically, accurately, easily and quickly without costly equipment. This preliminary study indicates that the MI could be an effective indicator of general health, except under conditions that may alter the value of the MI, such as special diets and medications.

**Electronic supplementary material:**

The online version of this article (doi:10.1186/s13104-016-2296-8) contains supplementary material, which is available to authorized users.

## Background

*S*-Adenosylmethionine (SAM, SAMe, or AdoMet) is a naturally occurring compound found in all living cells. SAM is a critical metabolite derived from adenosine triphosphate (ATP) and methionine (Met) via methionine adenosyltransferase (MAT EC2.5.1.6). SAM is an essential, metabolically pleiotropic molecule that participates in multiple cellular reactions as the precursor for the synthesis of glutathione and the major methyl donor for the methylation of nucleic acids, phospholipids, histones, biogenic amines, and proteins. SAM is the key molecule in the Met cycle and plays important roles in transmethylation, transsulfuration and aminopropylation. Methylation processes are the central biochemical basis of the neuropsychiatry of folate and B12 metabolism. The de novo synthesis of Met requires vitamin B12, which is involved directly in the transfer of the methyl group to homocysteine (HCys). Deficiency in either vitamin B12 or folate is considered the cause of low methylation index (MI). Metabolic hypomethylation might be one mechanism related to adverse clinical outcomes in B12-deficient individuals [1]. SAM provides the methyl group during the production of essential biomolecules such as carnitine (fat burner), acetyl-l-carnitine (neuro-nutrient, membrane-transporting agent), phosphocreatine (primary ATP reservoir), epinephrine/adrenalin (endogenous catecholamine, stress hormone and neurotransmitter), phosphatidylcholine (the most important membrane phospholipid), and melatonin (circadian rhythm modulator). Combined administration of *N-*acetyl cysteine (an antioxidant and glutathione precursor that protects against Abeta neurotoxicity), acetyl-l-carnitine (increases ATP levels, protects mitochondria, and buffers Abeta neurotoxicity), and *S*-adenosylmethionine (facilitates glutathione usage and maintains acetylcholine levels) enhances or maintains cognitive function and attenuates or prevents aggression, in mouse models of aging and neurodegeneration. Treatment with this nutraceutical combination can compensate for a lack of dietary folate and vitamin E [2]. Transsulfuration begins with SAH, the residual structure of SAM after donating the methyl group (trans-methylation). Hydrolysis of SAH yields HCys, which is converted to cystathionine, cysteine, and then glutathione, the hepatocellular antioxidant and life-saving detoxification agent. Aminopropylation is another process that is initiated by SAM through decarboxylation. Decarboxylated SAM is coupled to putrescine to generate spermidine and spermine, which are critical for cell growth, differentiation and DNA and RNA stability. Methylthioadenosine (MTA), the by-product of polyamine synthesis, is a powerful analgesic and anti-inflammatory agent that may be responsible for the clinical benefits observed in the treatment of osteoarthritis, rheumatoid arthritis and fibromyalgia with SAM [3, 4].

Defects in methylation lead to cell dysfunction and accompanying changes in SAM levels. Poor methylation or SAM deficiency has been implicated or related to the development of birth defects [5], cardiovascular disease [6], cancers [7], liver disease [8], and many other diseases [9–14, 18, 20, 21]. SAM levels have been identified as a diagnostic marker for *Pneumocystis carinii* pneumonia (PCP) in patients with immune-compromised conditions [15]. SAM has been used clinically for the treatment of liver disease, arthritis [16], and depression [17]. SAM produced cognitive improvement in patients with Alzheimer’s disease [19]. Treatment with SAM has been confirmed to be as effective as prescription tricyclic antidepressants [22] and non-steroidal anti-inflammatory drugs (NSAIDS) [3], with efficacy for the treatment of some liver conditions, such as cholestasis in pregnancy and intrahepatic cholestasis associated with liver diseases [23, 24]. More significantly, SAM is well tolerated, and no serious side effects have been observed. SAM provides rapid relief and is superior to tricyclic antidepressant treatment [25].

A deficiency of the major dietary sources of methyl donors, Met and choline, leads to the formation of liver cancer in rodents [26]. Extra-hepatic tumor formation increased in carcinogen-treated animals fed diets low in methyl donors including Met, choline, and folic acid [27]. Several mechanisms explain the enhancing effects of dietary methyl deprivation on carcinogenesis [28], of which the most accepted is that dietary methyl deficiency results in abnormal DNA methylation [29]. A critical metabolite in this hypothesis is SAM, the sole methyl donor in the body. Studies have linked deficiencies in folic acid, vitamin B12, and Met with an increased risk of cancer in various organs [30]. The question thus arises whether such deficiencies exert their activities through diminished availability of SAM. Studies have also demonstrated the utility of determining erythrocyte SAM and SAH and of plasma HCys in assessing disease status [31].

Due to the importance of SAM and SAH, an easy and reliable method is needed to measure their concentrations in a biological sample. A classic method for measuring SAM and SAH in rat liver utilizes the tripolyphosphatase activity associated with SAM synthetase in rat liver [32]. Tripolyphosphatase activity is stimulated by low concentrations of SAM [33]. The reported sensitivity of this method is 0.1 nmol of SAM in an assay volume of 0.1 mL (i.e., 10^−6^ M). The samples are lyophilized, homogenized in acid, and centrifuged. The supernatant is then passed through Dowex 1 to remove endogenous inorganic phosphate and other potential interferons in the tissue. Great care is needed to avoid inorganic phosphate contamination from all reagents, including the enzyme preparation and glassware. The disadvantages of this assay are a clear lack of specificity, low sensitivity (1 µM), and difficulties related to controls and comparisons among assays in different laboratories. Another common method for measuring SAM in tissues or biological fluids is HPLC or electrophoresis after sample preparation, which normally comprises protein precipitation and/or extraction [34]. Post-column detection may include derivatization followed by measurement by absorption, fluorescence, or electrochemical changes and, more recently, by LC–MS/MS [35]. These methods are laborious, time-consuming and require expensive equipment. LC–MS/MS does not consider the biological relevance of the detected metabolites. Therefore, the use of LC–MS/MS to measure SAM and SAH may not be accurate or complete from a biological perspective. Chemical methods can only detect the free form of SAM or SAH at the time of sample collection and exclude any SAM or SAH associated with other biomolecules. The inability of LC–MS/MS to detect SAM molecules that fall within the specific molecular weight range defined by LC–MS/MS in a sample does not indicate that SAM has been completely degraded, lost or is not functional. Furthermore, the SAM standard used to train LC–MS/MS is not identical to SAM from living cells. However, for a technology such as LC–MS/MS, a molecule identical to the molecule of interest is required as the training standard. Recent studies have suggested that GC–MS and LC–MS analytical methods may not accurately measure metabolites due to changes caused by lengthy and high-temperature manipulation processes during sample extraction, preprocessing and measurement [36].

SAM is an intrinsically unstable molecule, and its optical density maximum of 258–260 nm is not unique. Thus, the determination of the concentration of SAM in various biological fluids and tissues is a challenging task. A simple, convenient method that does not require costly instrumentation is clearly desirable for determining the biological concentration of SAM and monitoring changes in SAM levels in body fluids, tissues and organelles. The MI is defined as the ratio of the concentration of SAM to the concentration of SAH. Given the important roles of SAM and SAH in various pathological processes, it is desirable to conveniently measure the levels of SAM and SAH using methods that can be performed in typical research and clinical laboratories. The availability of specific antibodies against SAM and SAH has enabled the development of various forms of immunoassays to satisfy different needs. In this study, the generation and characterization of anti-SAM and anti-SAH antibodies as well as their applications are described. The usefulness of blood SAM and MI as markers for evaluating the roles of SAM in the onset and development of human diseases was also evaluated. This investigation included the determination of SAM, SAH, and MI in blood samples collected from normal and diseased subjects.

## Methods

### The hapten used to generate anti-SAM antibodies

Many SAM analogs were synthesized as shown in US patent No. 8,344,115 and evaluated to identify the best analog to generate the best anti-SAM antibodies. One of the most promising analogs, 5′-*N-*methyl, 5′-*N-*butyryl-5′-deoxyadenosine, or 5′-((3-carboxypropyl)methylamino)-5′-deoxy-adenosine, abbreviated as aza-deamino-SAM or shortened as aza-SAM, was employed to immunize Balb/c mice. All asymmetric carbons in aza-SAM maintained their chirality.

### Preparation of KLH-aza-SAM

To a flask containing 15 mg of aza-SAM that was vacuum-dried overnight, appropriate amounts (proportional to aza-SAM) of DCC (N,N′-dicyclohexylcarbodiimide), NHS (*N-*hydroxysuccinimide) and KLH (keyhole limpet hemocyanin) were added. Approximately 1.5 mL of dry DMF (dimethylformamide) was then added to the flask. The solution was stirred at room temperature. Formation of the NHS ester was confirmed by thin layer chromatography. The final volume after dialysis was 29.5 mL with a concentration of 0.6 mg/mL.

### Preparation of PLL-aza-SAM (poly-l-lysine-conjugated aza-SAM)

A 4.72-mg quantity of aza-SAM was dissolved in 1 mL of DMF with 6.5 mg EDC (1-ethyl-3-(3-dimethylaminopropyl) carbodiimide. Next, HCl and 4 mg of NHS were added, followed by stirring at room temperature in the dark overnight. Then, 1.5 mg of PLL was dissolved in 1 mL of 10 mM PBS pH 8.2. Aza-SAM was then added slowly to the PLL solution, and the mixture was incubated overnight in the dark. The reaction mixture was dialyzed for 48 h against 10 mM PBS, pH 7.3.

### Preparation of BSA-SAH

A 3.8-mg quantity of SAH (Sigma) was dissolved in 1.5 mL of PBS. Then, 10 mg of EDC HCl (Sigma) and 4.5 mg of NHS were added, and the mixture was stirred at room temperature for 24 h. We then dissolved 12.9 mg of BSA in PBS. The SAH was added to the BSA solution, and the mixture was incubated at 4 °C in the dark overnight.

### Preparation of HRP-conjugated antibodies

To develop a more efficient ELISA, HRP (horseradish peroxidase, Sigma) was directly conjugated to mouse anti-SAM and mouse anti-SAH antibodies. Five milligrams of HRP (dissolved in deionized water at 10 mg/mL) was added to 0.5 mL of freshly prepared 0.06 M sodium periodate and incubated at 4 °C for 30 min. Approximately 5 mg of antibody was added to the HRP solution and dialyzed overnight against 50 mM carbonate buffer, pH 9.6. Approximately 2 mg of sodium borohydride was added to the reaction mixture and incubated in the dark at 4 °C for 2 h with shaking once every 30 min. The sample was then dialyzed against 0.01 M PBS, pH 7.2, for at least 18 h.

### Procedures for generating monoclonal antibodies against SAM and SAH

Mouse monoclonal antibodies were produced based on procedures developed in a previous work [37]. Immunization was performed via subcutaneous injection of 0.1 mg of antigen at multiple sites into Balb/c mice. The initial injection consisted of a 1:1 mixture of complete Freund’s adjuvant and aza-SAM-KLH or SAH-BSA conjugate solutions in PBS upon emulsification. The subsequent 3 injections were similar except for the use of incomplete Freund’s adjuvant. Blood was collected from each immunized mouse at 1–2 weeks. The obtained antisera were then evaluated to determine the immune response and the antibody titer. Mice with satisfactory titers were primed via intravenous injection with immunogen three days prior to its sacrifice. The spleens of the mice were harvested and homogenized. The spleen cells were then fused with myeloma SP2/0 cells, and the fused cell suspension was plated out in 96-well microtiter plates. Hybridomas were grown in RPMI 1640 medium enriched with 18% fetal bovine serum, HAT and HT supplements and then screened. Clones that were positive for SAM or SAH were selected for further studies, including cross-reactivity tests. After the clones were obtained, the cells were injected into mice for ascite production. The antibody was then purified from the ascites using a protein A affinity column.

### Methionine adenosyltransferase (MAT)-catalyzed SAM biosynthesis

Solutions of 1 mM ATP and L-Met (Sigma) were prepared in 100 mM Tris, 100 mM KCl, 20 mM MgSO_4_, and 1% ProClin, pH 7.42. Recombinant *E. coli* MAT was added to the above substrates at a concentration of 0.6 mg/mL or 1 mg/mL, respectively. The reaction mixture was analyzed in duplicate using the SAM cELISA described in this section. The synthesized SAM was measured every 10 min after incubation at 37 °C for 20–80 min.

### Blood sample collection

Informed consent for sample collection and publication of clinical data was obtained from the participants. Sera were obtained from 81 normal volunteers, 310 plasma samples from the Changsha Blood Center and sera from 505 patients. For the plasma samples, peripheral venous blood was collected in tubes with EDTA and mixed well. The tubes were immediately cooled to 4 °C and centrifuged at 2000 g or higher for 15 min within 30 min after blood collection to obtain plasma. The plasma was either used for the measurements or frozen at −20 °C for future use. Serum samples were collected in serum separating tubes and placed in the refrigerator for approximately 2 h until blood coagulation occurred. The remaining steps were similar to those used for plasma collection. Plasma from the blood center was stored in the refrigerator for 1–7 h before centrifugation and freezing.

The Supplemental Data (Additional file 1) provides details regarding disease information. Patient samples were collected from clinical laboratories with no further information about the patient and disease conditions, status and treatments. Statistical analyses were performed using R by Dr. Huaitian Liu (National Cancer Institute, National Institute of Health, Rockville, MD) and Sydney Wong (University of California, Berkeley, CA).

### Competitive ELISA (cELISA)

To quantify plasma SAM and SAH levels, direct cELISA was developed by immobilizing antigens to a solid plate first, followed by the addition of SAM or SAH and HRP-labeled anti-SAM or anti-SAH antibodies. Antigens from a sample or standards competed with the corresponding fixed amount of immobilized antigens for binding to specific HRP-labeled antibodies. The final HRP substrate absorption values at 450 nM were inversely proportional to the amount of detected antigen, i.e., the higher the OD450 value, the lower the level of SAM or SAH in a sample. The wells of a 96-well ELISA plate (Corning high-affinity strips for ELISA) were coated with 0.05 μg/mL PLL-aza-SAM or 0.5 μg/mL BSA-SAH at 4 °C overnight. A series of SAM or SAH standards (aza-SAM from 0 to 960 nM and SAH-Na from 0 to 100 nM) and samples were added, followed by a 1:30,000 dilution of HRP-anti-SAM to a final volume of 100 μl. The plate was sealed and incubated at 37 °C for 60 min. The plate was then washed 3 times with PBST (10 mM PB, 150 mM NaCl, pH 7.4, 0.1% Tween 20). TMB (3,3′,5,5′-tetramethylbenzidine) was added to the wells (InnoReagents), and the plate was sealed and incubated for 15 min at 37 °C. The reaction was then stopped, and the OD450 values were read using a Multiskan FC reader (Thermo Fisher). The standards were generated using heat-inactivated pooled normal human plasma to minimize the matrix effect.

### Other materials

All analogs used in the cross-reactivity tests were from Sigma.

## Results and discussion

### Antibodies and immunoassays

#### Characterization of the anti-SAM monoclonal antibodies 118-6 and 84-3

**Fig. 1:**
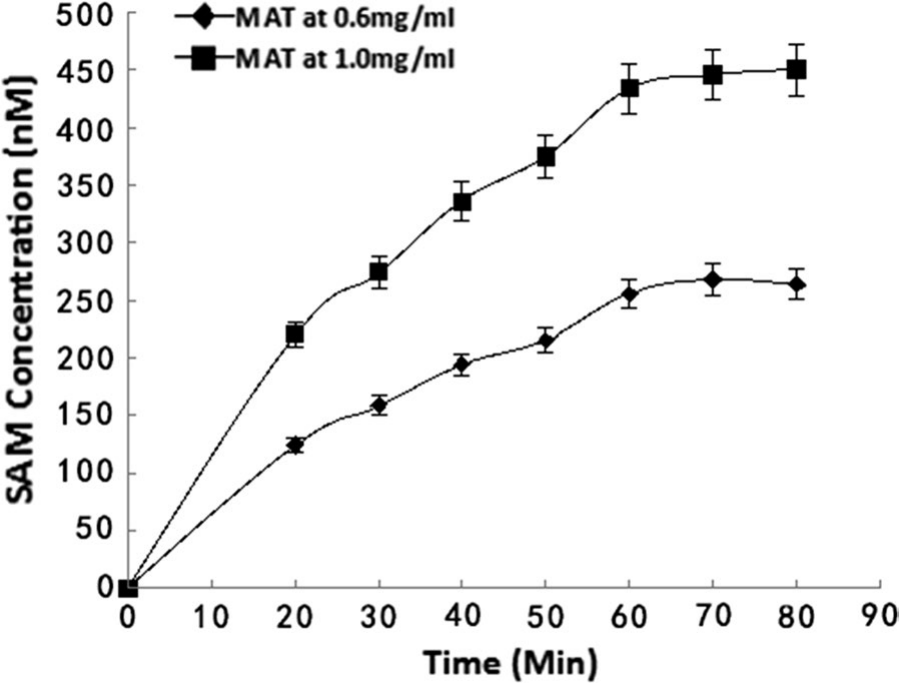
Detection of SAM synthesized from Met and ATP as substrates by the MAT-catalyzed reaction over time. The cELISA was used to measure SAM. The samples for the cELISA were the product of the following biochemical reaction. MAT was added to a maximum of 2 mM Met and ATP in the buffer described in the Methods at 37 °C. The biochemical reaction and ELISA were performed simultaneously and then stopped at 20, 30, 40, 50, 60, 70 and 80 min after the reaction. Different amounts of MAT and buffers were tested

**Fig. 2:**
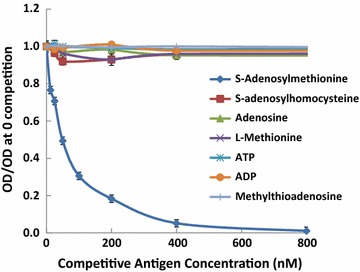
Cross-reactivity of the mouse anti-SAM monoclonal antibody 118-6 with SAM analogs. Each well of a 96-well plate was coated with 0.1 µg/mL PLL-aza-SAM, and serial dilutions of the SAM standard aza-SAM, *S*-adenosylhomocysteine (SAH), adenosine (Ade), L-methionine (Met), methylthioadenosine (MTA), adenosine diphosphate (ADP), and adenosine triphosphate (ATP) and 1:35,000 antibody were added. HRP-conjugated goat anti-mouse IgG antibody and substrate TMB were then added. The y-axis shows the OD450 of each well in relation to the wells without antigen competition. The x-axis shows the concentration of SAM. A lower value on the y-axis indicates greater competition

A good and useful antibody should have high specificity, sensitivity and affinity. The best way to demonstrate that the newly developed anti-SAM antibody specifically binds to SAM is to confirm that the anti-SAM antibody can bind to in vitro-synthesized SAM identical to the SAM synthesized in living cells. Figure 1 shows that the amount of SAM synthesized increased with a longer reaction time but reached a maximum after 60 min. This result indicates that the product of Met catalysis of ATP and Met was able to competitively bind the anti-SAM antibody. Dosage-dependent competition was detected as the sample was added to the cELISA system; SAM from the sample competed with the coated SAM hapten to bind the HRP-conjugated antibody 118-6. The sample consisted of Met, ATP and MAT in 100 mM Tris, 100 mM KCl, 50 mM MgCl_2_, pH 7.42, and 1% ProClin. The results indicated that the developed anti-SAM all specifically bind to physiologically produced SAM. The system permits simultaneous MAT-catalyzed SAM synthesis and SAM immunoassay, thus ensuring the accuracy of the results because SAM is unstable and termination of the MAT catalytic reaction at a precise time point is difficult. The use of cELISA to directly measure SAM in this scenario has even more important advantages for two reasons: (a) following synthesis, SAM remains associated with MAT for a period of time before release, and the traditional HPLC and MS methods for measuring SAM are unable to measure SAM in the MAT-SAM complex; (b) the preprocessing steps for HPLC and MS inevitably cause some SAM decomposition, leading to inaccurate results. The results in Fig. 2 indicate not only that the naturally produced SAM can compete in a dose-dependent manner with the SAM or aza-SAM coated onto the microtiter plates in terms of anti-SAM antibody binding but also that the method is a quick, easy and accurate way to measure the enzymatic activity of MAT. Thus, anti-SAM antibodies can be used to accurately measure MAT activity.

Cross-reactivity with SAM analogs was tested using up to 100-fold higher dosages of analogs than that of SAM antigen with the established cELISA method. Figure 2 shows a cELISA using the anti-SAM monoclonal antibody 118-6. In this assay, 0.1 µg/mL PLL-aza-SAM was used to coat a 96-well plate. Serial dilutions of Aza-SAM, SAH, adenosine, L-Met, ADP (adenosine diphosphate), ATP, MTA and 1:35,000 of 118-6 antibody were added. HRP-conjugated goat anti-mouse IgG antibody and TMB substrate were used to develop the results. The cross-reactivity of antibody 84-3 was similar to that of 118-6. At concentrations exceeding 10 μM Met, SAH, MTA, adenosine, ADP, and ATP, competition for the coated antigen (PLL-aza-SAM) with the HRP-antibody did not occur (data not shown). No competitive inhibition of the HRP signal was observed using these analogs, whereas inhibition was clearly evident when SAM was added at a much lower dosage than the analogs. These data indicate that the cross-reactivity of all analogs was <1%.

When the antibodies were adjusted to 1 mg/mL in an indirect ELISA assay, the titer of the anti-SAM monoclonal antibodies ranged from 1:10,000 to 1:100,000 depending on the criteria and conditions used.

To demonstrate the ease and affinity of antigen and antibody binding, ELISA assays were performed using different amounts of coated antigens. For the anti-SAM clone 118-6, when antigen was coated at a higher concentration of 0.2 μg/mL, half the maximum OD was obtained at a dilution of 1:54,800. The corresponding antibody concentration was (Ab) = (1 mg mL^−1^/160,000 g mol^−1^)/54,800 = 1.14 × 10^−10^ M, where 160,000 is the molecular weight of the antibody. When the coating antigen concentration was less than 0.1 μg/mL, half the maximum OD was observed at 1:44,300. The corresponding antibody concentration was (Ab)_t_ = (1 mg mL^−1^/160,000 g mol^−1^)/44,300 = 1.41 × 10^−10^ M, where n = 0.2/0.1(μg/mL) = 2, Ka = (n − 1)/2*(n(Ab) − (Ab)_t_) = 5.75 × 10^9^ L/mol = 1.74 × 10^−10^ M. For clone 84-3, when the coating antigen was 0.1 μg/mL, half the maximum OD was observed at approximately 1:13,0000. When the coating antigen was 0.05 μg/mL, half the maximum OD was detected when 84-3 was diluted at approximately 1:70,000. Using the same method as above, Ka = (n − 1)/2*(n[Ab] − [Ab]_t_) = 7.29 × 10^10^ L/mol = 1.37 × 10^−11^ M.

**Fig. 3:**
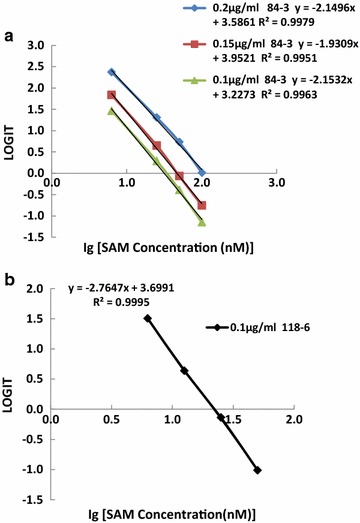
Standard curves for the mouse monoclonal antibody against SAM in the sensitivity tests. **a** Using antibody 84-3 at 1:35,000 with different amounts of coating antigens (0.1, 0.15, and 0.2 µg/mL) in an indirect cELISA. **b** Using antibody 118-6 at 1:32,000 with coating antigen at 0.1 µg/mL in an indirect cELISA. The x-axis shows the values of the base 10 logarithm of the standard concentrations. The y-axis is expressed as LOGIT, which is defined as Ln(A/A_S0_/(1 − A/A_S0_)), where A is the OD450 value of the standard and A_S0_ is the OD450 value of the blank (without antigen). The equation for each standard curve is shown

Tables 1, 2 shows the results of a SAM cELISA using three different coating amounts of PLL-aza-SAM when mouse anti-SAM antibody 84-3 was used at a dilution of 1:35,000 and from one coating plate when antibody 118-6 was tested in indirect cELISA assays. Different amounts of coating antigen, antibody, and buffer provide slightly different minimum detection limits. The data in Table 1 demonstrate that the minimum detection limits were between 1.6 and 3 nM when antibody 84-3 was used, depending on the coating antigens (see Table 1; Fig. 3a), and 7.8 nM when antibody 118-6 was used at a dilution of 1:32,000 (see Table 2; Fig. 3b). These values were estimated from the corresponding concentrations calculated from the standard curve equation given the OD450, that is, the average of the OD450 minus twice the standard deviation (SD) of the blank wells (without antigen). When the same amount of coating antigen (0.1 µg/mL) was used, antibody 84-3 exhibited higher sensitivity than antibody 118-6, consistent with the greater binding affinity of 84-3 compared with 118-6. The single-digit nM sensitivity indicates great potential in applications evaluating these critical metabolites under various conditions. Because SAM levels decreased under pathological conditions, it will be very important to obtain antibodies that are sufficiently sensitive to measure the low levels of SAM in samples.

**Table 1:** OD450 measured using different amounts of coating antigens with antibody 84-3

SAM standard (nM)	0.2 µg/mL			PLL-aza-SAM 0.15 µg/mL			0.1 µg/mL		
Blank	0.0498	0.056	0.0522	0.0476	0.0471	0.0482	0.0754	0.0515	0.0476
0	1.4709	1.4344	1.4072	1.215	1.2484	1.2651	1.0844	1.1231	1.0633
6.3	1.365	1.3257	1.2673	1.0789	1.0734	1.0842	0.879	0.9122	0.8995
25	1.1769	1.1498	1.1015	0.8231	0.844	0.8334	0.6209	0.6561	0.6679
50	1.0230	0.9856	0.9577	0.6428	0.6362	0.6005	0.4517	0.4555	0.5187
100	0.7687	0.7532	0.7292	0.4292	0.4203	0.4427	0.3036	0.3103	0.3071
Sensitivity^a^	3.0 nM			1.7 nM			1.6 nM		

^a^Sensitivity was estimated by the corresponding concentration calculated from standard curve equation given the OD450 (=OD450 at zero antigen − 2 × standard deviation of blank wells). ELISA was performed in triplicates. Mouse anti-SAM antibody 84-3 was used at 1:35,000

**Table 2:** Sensitivity of mouse anti-SAM antibody 118-6

SAM standard (nM)	OD450	OD450	OD450	Mean	SD
Blank	0.0556	0.0808	0.0616	0.0660	0.0131
0	1.2189	1.2831	1.3030	1.2683	0.0439
6.3	1.110	1.0648	0.9757	1.0502	0.0683
12.5	0.885	0.8004	0.8737	0.8530	0.0459
25	0.6252	0.6435	0.6108	0.6265	0.0164
50	0.3838	0.3995	0.3782	0.3872	0.0110
Sensitivity^a^	2.6 nM				

^a^Sensitivity was estimated by the corresponding concentration calculated from standard curve given the OD 450 (=OD450 at zero antigen – 2 × standard deviation of blank wells). ELISA was performed in triplicates. PLL-aza-SAM was coated at 0.1 µg/mL and the 118-6 used at 1:32,000

#### Characterization of the anti-SAH monoclonal antibody 301-1

**Fig. 4:**
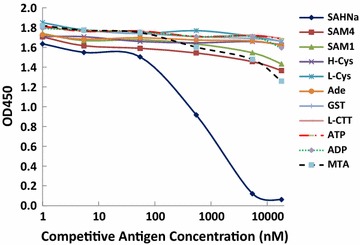
Cross-reactivity of the mouse anti-*S*-adenosylhomocysteine monoclonal antibody 301-1 with SAH analogs. Each well of a 96-well plate was coated with 0.5 µg/mL BSA-SAH. Serial dilutions of SAH standard (SAH sodium, SAH-Na), *S*-adenosylmethionine (SAM4: from Sigma-Aldrich Cat# A2408, SAM1: aza-SAM), homocysteine (H-Cys), l-cysteine (L-Cys), adenosine (Ade), glutathione (GST), L-cystathionine (L-CTT), methylthioadenosine (MTA), ADP (adenosine diphosphate), ATP (adenosine triphosphate) and properly diluted 301-1 were added. HRP-conjugated goat anti-mouse IgG antibody and TMB substrate were added to develop the color, and the OD450 was measured. The y-axis shows the OD450 for each test. The x-axis shows the concentration of SAH. A lower OD450 value indicates greater competition

The antibody specificity was evaluated by examining the cross-reactivity of 301-1 with SAH analogs. Figure 4 shows a cELISA using 301-1. A BSA-SAH concentration of 0.5 µg/mL was coated in each well of a 96-well plate. Serial dilutions of the SAH standard (SAHNa), *S*-adenosylmethionine (SAM4: Sigma Cat# A2408, SAM1: aza-SAM), HCys, l-cysteine (L-Cys), adenosine (Ade), glutathione (GST), L-cystathionine (L-CTT), MTA, ADP, ATP and properly diluted 301-1 were added. The cross-reactivity was approximately 1.5% with SAM, approximately 5% with MTA, and <1% with all other analogs. The relatively higher cross-reactivity with SAM and MTA (<5%) is not a concern because the physiological levels of SAM and MTA are much lower than 1 µM, a level at which no cross-reactivity with the antibody was observed.

Using the same method described above for affinity, when the coating antigen was 1 μg/mL, half the maximum OD was observed at a dilution of 1:1900. When the coating antigen was 0.5 μg/mL, half of the maximum OD was observed at approximately 1:1100. Thus, Ka = (n − 1)/2*(n[Ab] − [Ab]_t_) = 2.79 × 10^8^ L/mol = 3.6 × 10^−9^ M. The titer of 301-1 was in the range of 1:4000–8000 when the antibodies were adjusted to a concentration of 1 mg/mL in an indirect ELISA assay.

Table 3 shows the cELISA results for 0.5 μg/mL BSA-SAH coated onto a microtiter plate with mouse anti-301-1 at a dilution of 1:1000. Different amounts of coating antigen, antibody, and incubating buffer may provide slightly different minimum detection limits. The same method described above was used to calculate the minimum detection limit, which was approximately 15.6 nM.

**Table 3:** SAH standard curve and sensitivity of antibody 301-1

SAH (nM)	OD450	OD450	OD450	Mean	SD
250	0.4116	0.4426	0.442	0.4321	0.0177
125	0.5421	0.6358	0.5372	0.5717	0.0555
62.5	0.6055	0.7115	0.6444	0.6538	0.0536
31.3	0.7285	0.8199	0.7545	0.7676	0.0471
15.6	0.8045	0.8273	0.7975	0.8098	0.0156
7.8	0.7602	0.8113	0.7708	0.7808	0.0270
3.9	0.7722	0.9144	0.7786	0.8217	0.0803
0	0.8770	0.8992	0.8329	0.8697	0.0337
Sensitivity^a^	~15.6 nM				

^a^Sensitivity was estimated by the corresponding concentration calculated from standard curve given the OD 450 (=OD450 at zero antigen – 2 × standard deviation of blank wells). ELISA was performed in triplicates. BSA-SAH was coated at 0.5 µg/mL and mouse anti-SAH antibody 301-1 was used at 1:2000

#### cELISA

**Fig. 5:**
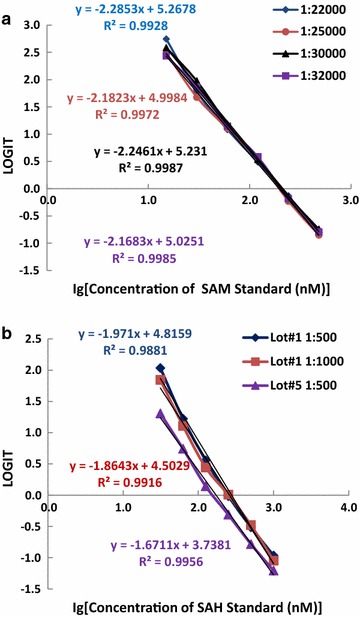
Standard curves for the quantification of SAM (**a**) and SAH (**b**) by the direct cELISA. The x-axis shows the values of the base 10 logarithm of the concentrations of the standards. The y-axis is expressed as the LOGIT, which is defined as Ln(A/A_S0_/(1 − A/A_S0_)), where A is the OD450 value of the standard and A_S0_ is the OD450 value of the control (without antigen). **a** HRP-118-6 was diluted 1:22,000, 1:25,000, 1:30,000 and 1:32,000. The standard curve equations for the 3 different diluted tests are shown in the figure. **b** Different lots of HRP-301-1 and different dilutions were used. The standard curve equations for the 3 tests are shown

The standard curves for cELISA of SAM and SAH are shown in Figs. 5a, b, respectively. LOGIT = Ln(A/A_S0_/(1 − A/A_S0_)), where A is the OD450 value of a sample or the standard and A_S0_ is the OD450 value of the control (without antigen). A negative LOGIT value indicates that A/A_S0_ is less than 50% and the inhibition rate (1 − A/A_S0_) is greater than 50%. As shown in Fig. 5a, the standard curves were very similar when different dilutions of HRP-anti-SAM antibody 118-6 of between 1:22,000 and 1:32,000 were used. As shown in Fig. 5b, different batches of HRP-anti-SAH antibody 301-3 provided slightly different competitive inhibition results, with Lot# 5 exhibiting greater inhibition. Competitive inhibition results in reduced OD450 readings from TMB substrates due to the addition of a high dosage of free SAM or aza-SAM, allowing less coated PLL-aza-SAM to bind to the HRP-anti-SAM antibody. After washing the microtiter plate, less HRP-anti-SAM remained on the plate, and thus the weaker OD450 was equivalent to an inhibitory effect. As shown in Fig. 5a, a significant portion of the SAM standard curve was within the negative portion of the y-axis (LOGIT). A negative LOGIT indicates competitive inhibition exceeding 50%. For the SAH standard curve, as represented in Fig. 5b, a small portion of the SAH standard curve was within the negative LOGIT. Therefore, the inhibition rate is much higher in SAM cELISA than in SAH cELISA, which is partly determined by differences in the affinity of the anti-SAM and anti-SAH antibodies.

SAH is normally low in humans, and thus the ability to measure low levels of SAH is essential. The sensitivities of the anti-SAM and anti-SAH antibodies reported herein are the best to date. Using direct cELISA, the minimum detection limit was approximately 2 nM for SAM and 15 nM for SAH. Other more sensitive immunological technologies can be developed to further reduce the minimum detection limit, increase the range of detection, or improve the assays via faster, easier and higher throughput and better quality control and precision. Sensitivity is essential for most IVD (in vitro diagnosis) applications.

SAH is metabolically linked to SAM, and the structure of SAH contains one less carbon (a methyl group) than SAM. The co-existence and structural similarity of SAM and SAH present a great challenge in the development of a method for the specific determination of the concentration of either molecule in a biological sample. The unstable nature of SAM further complicates its determination. As the immediate precursor of HCys produced in the body, SAH has recently been suggested to be a more sensitive indicator of the risk of vascular diseases than plasma HCys [38]. The total plasma concentration of SAH is normally much lower than that of HCys. As with SAM, SAH has no distinct absorption characteristics, hindering its detection in serum or plasma. Because SAH is the product of all methylation reactions involving SAM as a methyl donor, an increased concentration of SAH in tissues is frequently accompanied by a decreased concentration of SAM. Therefore, the ratio of SAM to SAH is considered a more sensitive indicator than the concentration of either SAM or SAH alone, particularly when their changes are subtle during the early stages of dysfunction or under abnormal conditions. In this work, we established convenient immunoassays to measure the MI quickly without costly instrumentation.

### The level of SAM varies with age and gender

#### Normal distribution of SAM and SAH levels in normal serum or plasma samples

**Fig. 6:**
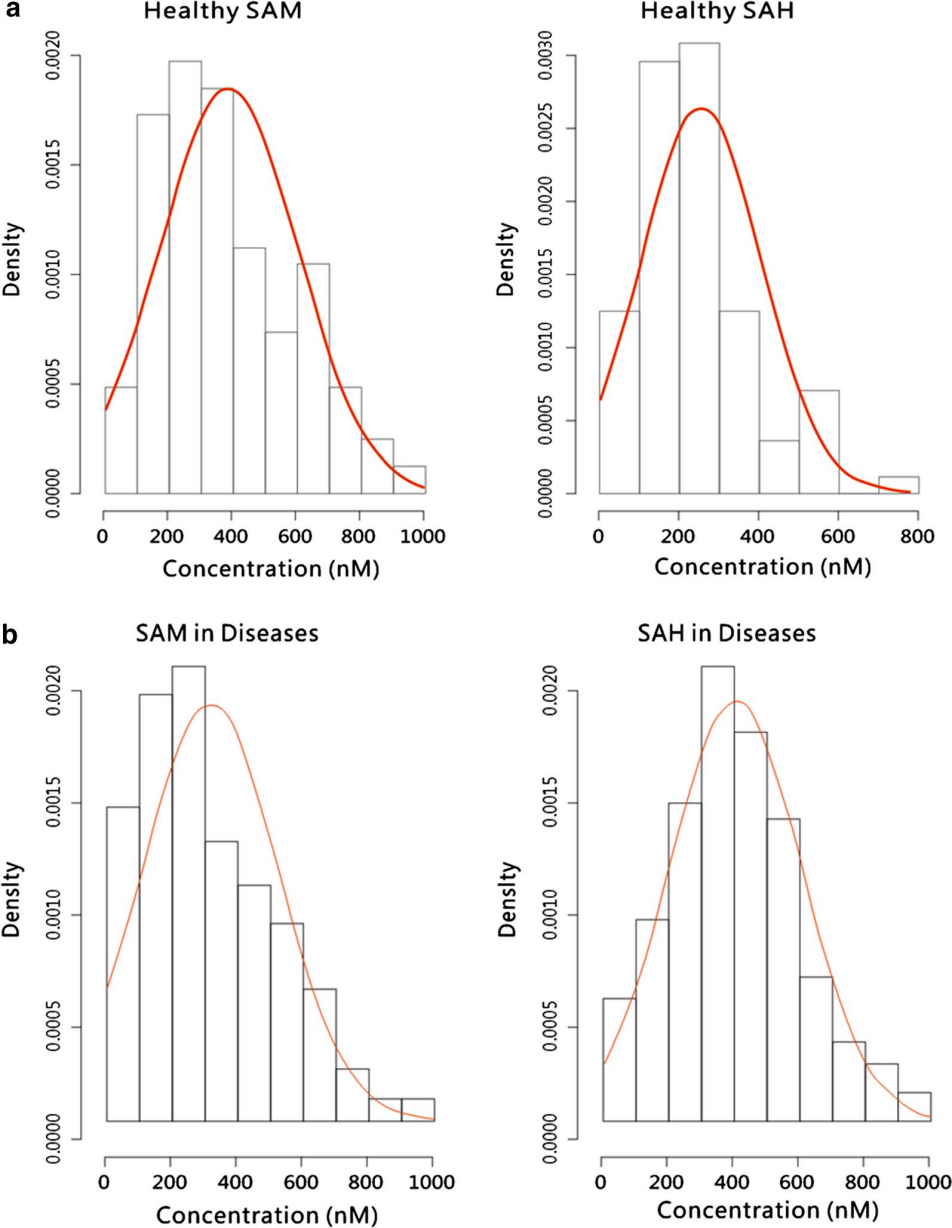
Distribution and values of SAM and SAH calculated using R programming. **a** 81 normal serum samples; **b** 291 diseased serum or plasma samples

Using the statistical methods in the R program, the distributions of SAM and SAH values among normal and diseased samples were calculated. Figure 6a shows the SAM and SAH values and distributions from 81 normal serum samples. The average and SD was 386 nM ± 216.2 for SAM, 256.9 ± 150.7 for SAH, and 2.2 ± 1.9 for the MI. Figure 6b shows the SAM and SAH values and distributions from 291 serum samples. The results suggest normal distributions of SAM and SAH values from the subjects investigated. Therefore, Student’s *t* test was employed for statistical analysis. The distribution graph of diseased SAM levels also indicated that a significant number of patients had SAM values less than 300 nM. In contrast to the graph of the diseased SAH cases, the mean value of SAM did not represent the values of the majority of the samples, partially implying that many factors affect the SAM level.

#### Distribution of normal plasma SAM levels in different genders and age groups

**Fig. 7:**
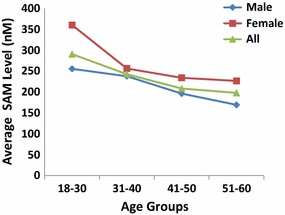
SAM levels in different age and gender groups. Data were obtained for 310 plasma samples from the blood center, which were obtained from 99 females and 211 males

In another study of 310 plasma samples from the blood center, the ranges, averages, and standard deviations of SAM levels in females and males were analyzed, and the case numbers and percentages in each group are summarized in Table 4. On average, women had higher SAM levels (296.92 nM) than men (232.86 nM). Furthermore, a larger percentage of females had SAM levels exceeding 240 nM than males (Table 4; Fig. 6), contributing to the higher average SAM levels determined in females. Table 5 shows the different ranges of SAM levels by age group. With increasing age, the percentage of people with SAM levels lower than 60 nM increased from 1.46% among those aged 18–31 years to 12.5% among those aged 51–60 years. However, with increasing age, the percentage of people with SAM higher than 240 nM generally decreased, except in the age group from 51 to 60 years. The decrease in SAM levels with age might be attributable to the following factors: too few cases in the 51- to 60-year-old age group; the 31- to 50-year-old group is subject to higher life stress and the greatest workload in China (a relatively larger percentage of people aged 31–50 years old were considered to have a sub-healthy condition, which might be related to lower SAM levels); a significant proportion of people aged 51–60 years are retired and most likely find ways to take good care of their health and are under less stress. The results shown in Table 5 and Fig. 7 indicate that women have higher average SAM levels than men (approximately 28% higher). This discrepancy is likely not due to differences in diet, geographical area or social status. The fundamental reasons for these differences are unknown and are likely related to metabolism and genetic background. SAM levels were higher in females than in males, consistent with another study of 81 normal serum samples. The differences in SAM and SAH levels between the 310 plasma samples and 86 serum samples may be attributable to the prolonged period that the plasma was stored in the refrigerator before freezing.

**Table 4:** Distribution of normal human plasma SAM levels by gender

SAM (nM)	Male no. (%)		Female no. (%)	
<30	0	0	1	1.01
30–60	9	4.2	3	3.03
60–120	44	20.85	9	9.09
120–240	70	33.18	33	33.33
240–480	75	35.55	39	39.39
480–960	13	6.16	14	14.14
>960	0	0	0	0
Avg. SAM	211	232.86	99	296.92
SD SAM	211	149.56	99	185.12

**Table 5:** Distribution of normal human plasma SAM levels in different age groups

SAM (nM)	Age 18–30 No. (%)		Age 31–40 No. (%)		Age 41–50 No. (%)		Age 51–60 No. (%)	
<30	1	0.73	0	0	0	0	0	0
30–60	1	0.73	5	6.10	4	5.33	2	12.50
60–120	17	12.41	16	19.51	17	22.67	3	18.75
120–240	42	30.66	28	34.15	30	40	3	18.75
240–480	58	42.34	28	34.15	20	26.67	8	50
480–960	18	13.14	5	6.10	4	5.33	0	0
>960	0	0	0	0	0	0	0	0
<60	2	1.46	5	6.10	4	5.33	2	12.5
>240	76	55.47	33	40.25	24	32	8	50
Total no.	137		82		75		16

Figure 7 shows the average SAM levels in different age and gender groups. In both genders, the SAM level continuously decreased with increasing age, indicating that SAM might be directly or indirectly involved in the aging process. Table 5 shows that a higher percentage of people older than 51 years had SAM levels of less than 60 nM, which indicates that older age was associated with decreased SAM levels. ANOVA analysis was performed with SAM, SAH and the MI as the response variables and age and gender groups as the explanatory variables. The p value was 0.0343 for the age group in terms of SAM (significant at level α = 0.05) and 0.0511 for the gender group in terms of SAM (significant at level α = 0.1). These results indicate that the decreasing SAM trends for age and gender, as shown in Fig. 7, are statistically significant but at different levels. The levels of SAM in females were significantly higher than those in males. In a normal population, the younger a person, the higher the level of SAM. The SAM level was significantly higher in the 18- to 30-year-old age group than in any other age group older than 31 years. The SAM level in children and youth younger than 18 years was not evaluated.

A previous report indicated global DNA hypomethylation in older individuals compared with a younger population. The data indicated that age-related variations in the global DNA methylation profile of leukocytes might be modulated by the daily intake of carbohydrates, lipids, vitamin B6, and magnesium and associated with serum protein levels. These nutritional factors may cause the SAM level to decrease greatly due to metabolic changes that occur as people age, contributing to the hypomethylation observed in the elderly population.

Men and women have different hormone levels and considerably different hormone compositions, and thus their metabolisms differ. Liver cells express estrogen receptors that are related to development, lipid metabolism and insulin sensitivity, hepatic carcinogenesis, protection from drug-induced toxicity and fertility. In addition, estrogens can modulate growth hormone activity in the liver by acting centrally (pituitary growth hormone) and by modulating the GHR-JAK2-STAT5 pathway [39]. Age and gender affect metabolic syndrome and metabolic disorders in all-cause and cardiovascular disease-related mortality [40]. The Met cycle may be related to the pathways indicated above, and further investigations may elucidate the gender-dependent SAM level. A recent clinical trial assessing the efficacies of SAM for the treatment of depression in female and male patients indicated that gender might impact the antidepressant efficacy of SAM, with a greater therapeutic effect observed in males [41] only. The average lower level of SAM in males than in females may contribute to the underlying mechanisms.

### SAM and MI are reduced in disease

#### Cancers

Table 6 (after the Reference section) Study A shows the results of statistical analysis of data from cancer samples compared with normal serum samples by t-test. In liver cancers, SAH levels did not change significantly, and there was no significant change in the MI. By contrast, all other cancers exhibited a significant decrease in SAM levels and a significant increase in SAH levels, leading to more significant decreases in the MI, as indicated by the number of asterisks (*) for the t-test results. The dynamics of the levels of SAM and SAH in liver appear to differ from those in other tissues and organs. Our prior investigations using immunohistochemistry and flow cytometry to assess normal and malignant liver cell lines indicated that in cancer cells, both intracellular SAM and SAH are significantly reduced. Although this observation may only reflect SAM and SAH levels during a particular stage in each case of pathogenesis, it supports the unique characteristics of the MI in the liver compared to other tissues and organs, probably because SAM is primarily metabolized in the liver. The differences in SAH levels in liver cancers from other tissues or organs emphasize the importance of SAM measurements for describing the severity of liver disease.

**Table 6:** Results of *t* test in diseases from different studies

Diseases	Response variable	Mean	*p* value	Significance
Study A				
Liver cancer	SAM	278.0652	0.03451	**
Liver cancer	SAH	293.35	0.3288	
Liver cancer	MI	1.562109	0.1131	
Lung cancer	SAM	262.5039	0.001248	***
Lung cancer	SAH	326.9877	0.007807	***
Lung cancer	MI	0.9710702	3.67E−06	****
Other cancer	SAM	283.3233	0.00116	***
Other cancer	SAH	394.7301	5.61E−06	****
Other cancer	MI	0.8156412	8.12E−09	****
Study B				
Cerebrovascular diseases	SAM	415.4277	0.6064	
Cerebrovascular diseases	SAH	363.3671	0.03015	**
Cerebrovascular diseases	MI	1.163449	2.03E−05	****
Depression	SAM	337.385	0.3531	
Depression	SAH	442.379	0.01176	**
Depression	MI	0.87051	4.81E−06	****
Parkinson’s disease	SAM	285.5726	0.07684	*
Parkinson’s disease	SAH	794.5792	0.06972	*
Parkinson’s disease	MI	0.74988	7.18E−06	****
Study C				
Cerebrovascular diseases	SAM	357.9406	0.4365	
Cerebrovascular diseases	SAH	353.175	0.001237	***
Cerebrovascular diseases	MI	1.148035	2.31E−05	****
Diabetes	SAM	262.3474	0.001136	***
Diabetes	SAH	372.0686	0.003831	***
Diabetes	MI	0.8619158	9.20E−08	****
High blood pressure	SAM	288.3645	0.0389	**
High blood pressure	SAH	358.1118	0.01378	**
High blood pressure	MI	0.9178064	3.93E−07	****
Heart diseases	SAM	315.962	0.07938	**
Heart diseases	SAH	440.9451	5.95E−07	****
Heart diseases	MI	0.7822701	5.61E−09	****
Inflammation	SAM	223.732	3.05E−06	****
Inflammation	SAH	292.012	0.2051	
Inflammation	MI	0.8193137	1.91E−08	****
Kidney disease	SAM	307.6073	0.1077	
Kidney disease	SAH	497.1204	7.76E−06	****
Kidney disease	MI	0.6976807	3.69E−09	****
Chronic liver diseases	SAM	356.3407	0.5037	
Chronic liver diseases	SAH	444.0027	1.22E−06	****
Chronic liver diseases	MI	0.8921861	2.72E−07	****
Respiratory diseases	SAM	393.3392	0.8827	
Respiratory diseases	SAH	486.5144	4.24E−08	****
Respiratory diseases	MI	0.7916811	5.27E−09	****

* Represents significance at significance level = 0.1, ** represents significance at significance level = 0.05; *** represents significance at significance level = 0.01, **** represents significance at significance level = 0.001

#### Liver diseases

In a controlled study conducted at Xiangya Hospital, SAM levels were measured in patients with severe liver conditions such as acute viral hepatitis, cirrhosis, and liver failure. Patients who received SAM as a liver-protecting medicine were excluded from the study. We observed that SAM levels and the MI in different types of liver diseases, including hepatitis, carcinoma, cirrhosis and liver failure, were very different from those in the normal population. These findings were striking. Patients with hepatitis were diagnosed with acute severe viral hepatitis (B and C). Patients with cirrhosis were also in an advanced stage requiring aggressive treatments in the hospital. Liver failure indicated that liver functions could no longer be compensated, and laboratory results revealed a severe loss of liver function. In hepatitis, 19.57% of the 46 samples had SAM levels higher than 120 nM, in contrast to 95% of normal samples; 4.35% had SAM levels greater than 240 nM, in contrast to 69.14% of the normal samples; and 94.87% of the 46 samples had an MI less than 0.5, in contrast to 8.64% of the normal samples. Among patients with hepatocyte carcinoma, 4 of 14 (28.57%) exhibited SAM levels greater than 120 nM, 7.14% had SAM levels greater than 240 nM, and 85.71% had an MI less than 0.5. Among patients with cirrhosis, 3 of 20 (15%) exhibited SAM levels greater than 120 nM, 5% had SAM levels greater than 240 nM, and 89.47% had an MI of less than 0.5. Finally, all 19 patients with liver failure had SAM levels less than 120 nM and an MI of less than 0.5. These results demonstrate that SAM levels and MI values were significantly reduced compared with those in the normal controls; thus, SAM levels could be used as a biomarker to assist in the diagnosis of liver diseases.

#### Brain diseases

Table 7 presents the SAM and MI values of normal subjects (two different studies) and 40 cases of brain disease. The percentages of cases with SAM levels greater than 240 nM was lower among cases of cerebrovascular disease, Parkinson’s disease and depression compared with normal subjects. Moreover, among the cases of brain disease, there was a significant increase in the percentage of patients with an MI of less than 0.5 (8.64% of normal people with MI < 0.5 from one study versus 20% of Parkinson’s disease and depressed patients), and the percentage of patients with Parkinson’s disease and depression patients with an MI greater than 1 was significantly lower than the percentage of normal subjects with an MI greater than 1. These findings suggest that MI might be a good marker of Parkinson’s disease and depression, with a lower MI indicating a higher probability of Parkinson’s disease or depression. The results of the t-test analyses of SAM, SAH and MI between normal and brain disease are shown in Table 6 Study B. The results demonstrated that SAM is decreased in cerebrovascular diseases, whereas the decrease in SAM in depression was not significant. However, the decreases in MI were very significant for all types of brain disease, suggesting that MI is a better biomarker for brain diseases.

**Table 7:** SAM levels and MI values in brain diseases

Diseases (case #)	% of SAM > 240 nM	% of SAM > 120 nM	% of MI > 2	% of MI > 1	% of MI < 0.5
Normal plasma (310)	90.36	96.79	6.95	42.47	16.98
Normal serum (81)	69.14	95.06	46.91	77.90	8.64
Cerebral hemorrhage (10), embolism (6), infarction (4)	85	90	0	65	0
Parkinson’s disease (10), depression (10)	70	90	0	25	20

#### Other diseases

Table 6 Study C shows the t-test results for other diseases versus normal serum controls. The decreases in SAM in cerebrovascular diseases, kidney diseases, chronic liver diseases, and respiratory diseases were not significant compared with the normal controls, whereas the increases in SAH in this diseases were significant. The levels of SAM decreased significantly in diabetes, high blood pressure, heart diseases and inflammation. However, the decreases in the MI were all extremely significant for all analyzed diseases, which suggests that the MI is a better biomarker for these diseases.

#### SAM and inflammation

Interesting results were obtained for the levels of SAM, SAH and MI under conditions of inflammation (Table 6 Study C). The levels of SAM were significantly reduced in inflammation conditions, whereas the increase in SAH in inflammation conditions was not significant. The diseases included in this inflammation group were mostly digestive, vascular and other benign inflammatory conditions. Common features of the group were chronic and widespread inflammatory responses. Thus, SAM appears to play a larger role in these conditions than SAH. This phenomenon is likely related to the important role of SAM in inflammatory processes in which internal strong anti-inflammatory factors such as spermine or spermidine or MTA are generated as a result of SAM aminopropylation. Under certain pathological conditions, SAM aminopropylation might be blocked or deficient due to reduced SAM levels, resulting in defective anti-inflammatory functions of SAM and uncontrolled inflammatory reactions. Accordingly, supplementation with SAM would, in most cases, control inflammation [42]. In North America, SAM pills are sold over-the-counter for the relief of joint pain or the treatment of osteoarthritis. The present study provided a foundation for the use of SAM for controlling inflammation.

#### Ranges of the MI

Table 8 summarizes the ranges and averages of the MI for all diseases in this study. The findings are consistent with the results of t-tests demonstrated that the MI is significantly higher in normal subjects (average value of 2.23) than diseased patients (average of 0.87 or <1.56). This clear difference between the normal and diseased groups is also apparent for the maximum MI. The MI value was between 4 and 6 in cancer, and therefore the average MI in cancer was relatively higher than that in other diseased groups, primarily due to higher SAM levels in the in cancer samples. This result is consistent with an article by Greenberg et al. [43], who reported elevated serum SAM levels in patients with lung cancer compared with smokers with benign lung disorders and healthy nonsmokers. No significant correlations were identified between SAM levels and tumor cell types, nodule size, or other demographic variables. The transient high SAM levels may be caused by the release of intracellular SAM from cells into the blood stream (as confirmed by IHC staining of SAM from cancer cells (data not shown) during certain stages of cancer progression). Thus, we observed wide ranges of MI values in all types of cancers. SAM release was significant only in certain stages of cancer development. The release of SAM into the blood stream at high levels contributes to the large ranges in SAM levels observed in all cancers. An MI of 5.67 was detected in only one of 68 cerebrovascular disease samples. All other MIs were less than 2.09. If that sample is considered an outlier, the average MI for this group is less than 1 rather than 1.06. Therefore, cerebrovascular diseases should be considered among other diseases in evaluating the range and average value of the MI.

**Table 8:** Ranges and averages of MI values in different diseases

Group	Range of MI	Average MI
Normal	0.40–6.50	2.23
All diseases	0.07–5.67	0.87
Cerebrovascular diseases	0.36–5.67	1.06
Parkinson’s Disease	0.08–1.86	0.75
Depression	0.36–1.94	0.87
Diabetes	0.11–3.86	0.86
HBP	0.10–1.19	0.92
Heart disease	0.07–1.95	0.78
Inflammation	0.28–2.13	0.82
Kidney disease	0.10–1.92	0.70
Liver diseases	0.22–2.50	0.89
Respiratory diseases	0.13–1.49	0.79
Other cancers	0.10–5.42	0.81
Liver cancer	0.13–4.98	1.56
Lung cancer	0.06–5.42	0.68

### SAM and the MI as disease biomarkers

If the antibodies used in the assays are specific, the MI measured by immunoassays carries greater weight than the MI measured based on free SAM and SAH alone. Because the free and complex forms of SAM and SAH are subject to enormous dynamics, a method that evaluates the complete profiles has obvious advantages over simply examining one subset. SAM, SAH and the MI were previously used as biomarkers for diagnosing acute rejection after renal transplantation and nephrotoxicity events [44]. The present work and other studies investigating *Pneumocystis carinii* pneumonia (PCP) in patients with HIV infection or other immunocompromised conditions [45] suggest that the measurement of SAM levels by cELISA could be an effective tool to diagnose the occurrence of PCP in immunocompromised conditions.

We evaluated SAM, SAH and MI levels in liver diseases in three different scenarios: (a) hepatocyte cancers (Table 6 Study A), (b) acute or severe liver diseases described in the liver disease section above, and (c) chronic liver disorders (Table 6 Study C). The results from (b) indicate that SAM and the MI can be used to identify severe liver diseases based on the significant decreases in these parameters. The cutoff value could be set between 120 nM and 240 nM. Because SAM is actively generated and metabolized in the liver, deterioration of liver function will significantly affect SAM metabolism. The results from (c) indicate that a non-significant decrease in SAM might be related to milder and chronic liver conditions in which SAM might be compensated as part of liver functional compensation over time. The results from (a) demonstrate a significant decrease in SAM in liver cancer patients but no significant increase in SAH levels, suggesting differences in the disruption of the Met cycle between carcinogenesis and other pathological processes. The level of SAM in hepatic carcinoma was decreased significantly, in contrast to a report by Li [46]. The transient high levels of SAM were caused by the release of intracellular SAM from cells into the bloodstream (confirmed by immunohistochemistry staining of SAM in cancer cells, data not shown) at some stage of cancer progression. Because SAM release is only significant in certain stages of cancer development, after this release is complete, the elevated SAM levels in the bloodstream will not be maintained, contributing to the large ranges of SAM levels observed for all cancer types Our samples were collected from various stages of cancers, and therefore wide ranges of MI values were observed in the cancer samples. Table 8 also shows that the average MI for liver cancers was much higher than that for other diseases, indicating that in some liver cancers, the levels of SAM were high, which is consistent with Li’s report. In conclusion, SAM is a biomarker of the stages or severity of liver diseases.

Although we did not exhaustively examine all disease types, the cases observed in the present study were derived from a variety of diseases, although some diseases were combined for analysis due to the small number of samples. The significant reduction of the observed in many disease types suggest that MI could serve as a useful biomarker for the evaluation of an individual’s health status. Furthermore, the diseased group exhibited significantly lower SAM and MI levels and significantly elevated SAH levels compared to healthy people. MI can thus serve as a health status indicator. We believe the major use of the MI as a health marker would involve monitoring changes in the MI of an individual over time. MI can be used as a screening biomarker to detect an unfavorable health condition in humans, although further examinations are warranted for a final disease diagnosis. Further clinical investigations in different races and segments of the population stratified by age, gender, diet, medication or even genetic background are needed. The MI will likely be useful for aiding the detection of disease conditions in healthy populations and be included in annual health examination panels.

## Conclusions

In the present study, monoclonal antibodies against SAM and SAH were characterized, and associated immunoassays were established. This preliminary investigation indicated that **S**AM, SAH and MI are good indicators of general health. In the absence of factors that may alter the value of the MI, such as special diets and medications, a significant reduction of the MI compared with an individual’s baseline MI reference profile may suggest unfavorable conditions or diseases and warrant a visit to the doctor.

## Additional file

**Additional file 1.** Information on cases collected from clinical labs.
