# Supplementary material for: Immunoassay of S-adenosylmethionine and S-adenosylhomocysteine: the methylation index as a biomarker for disease and health status
Source: BMC Res Notes. 2016 Nov 28;9:498. doi: 10.1186/s13104-016-2296-8 (PMC5127003; doi:10.1186/s13104-016-2296-8)
Supplement: Supplementary file 1 — Additional file 1. Information on cases collected from clinical labs. [file 13104_2016_2296_MOESM1_ESM.pdf]

Information on cases collected from clinical labs

Table 1a Disease and Case Information –Study C

Disease	Case No.
Cerebrovascular diseases + Parkinson's Disease	45 + 3
Diabetes	43
High Blood Pressure	22
Heart diseases	51
Inflammation	35
Kidney disease	26
Chronic Liver diseases	30
Respiratory diseases	36

Table 1b Cancer and Case Information - Study A

Cancer	Case No.
Bladder Cancer	2
Breast Cancer	3
Colon Cancer	16
Esophagus Cancer	3
Gallbladder Cancer	1
Lipoma	2
Liver Cancer	23
Lung Cancer	75
Lymphoma	2
Multiple Myeloma	1
Ovary Cancer	2
Prostate Cancer	5
Throat Cancer	4
Thymoma	1
Thyroid cancer	1
Uterus Cancer	5
Vascular Cancer	1
Cancer	27

Table 1c Brain Disease Information – Study B

Disease	Case No.
Cerebrovascular diseases (hemorrhage (10), embolism (6), infarction (4))	20
Depression	10
Parkinson's Disease	10

Inflammation: a collection of benign inflammatory responses and related diseases: Lower limb atherosclerosis obliterans, Infectious mononucleosis, Coagulopathy, Benign prostatic hyperplasia, Arterial embolism, Duodenal ulcer, Reflux esophagitis, Remnant gastritis, Acute gastric infarction, Acute gastroenteritis, Acute pancreatitis, Ankylosing spondylitis, Acute tonsillitis, Scrub typhus (scrub typhus), Chronic gastritis, Gastrointestinal bleeding, Warty stomach (antral), Hydrocele, Intestinal obstruction, Gastroenteritis, Cholecystitis, Pancreatitis, Lumbar disc herniation, Cellulitis, Hemophilia, Thrombocytopenic purpura, Hiatal hernia.

Heart diseases: Coronary heart disease, Chronic respiratory heart disease, Respiratory heart disease

Kidney diseases: Nephritis, Uremia, Chronic renal insufficiency, Urinary stones, Renal insufficiency, Hydronephrosis, Kidney stones, Ureteral stones, Staghorn calculi

Respiratory diseases: Asthma, Asthmatic Pneumonia, Chronic bronchitis, Chronic laryngitis, Chronic obstructive respiratory emphysema, Chronic obstructive respiratory disease, Silicosis respiratory fibrosis, Emphysema, Pneumonia, Tuberculosis

Cerebrovascular diseases**:** hemorrhage, embolism, infarction, Transient ischemic, Nerve root type cervical spondylosis, Meniere's syndrome, Cerebral arteriosclerosis, Sub-arachnoid hemorrhage

Chronic Liver diseases: Chronic hepatitis (virus B, C), Liver lesions, Obstructive jaundice, Alcoholic hepatitis, Fatty liver, Gall bladder disease.
